# A novel broad-spectrum lytic phage vB_EcoM_P3322: isolation, characterization, and therapeutic potential against avian pathogenic *Escherichia coli*

**DOI:** 10.3389/fcimb.2025.1645263

**Published:** 2025-09-22

**Authors:** Ying Huang, Mengfei Zhang, Yuan Tian, Xueqin Lan, Wanjing Jin, Yixin Bai, Qirui Zang, Mingshuai Chen, Zhanqiang Su, Wei Zhang, Gulina Aishan, Mingyang Geng, Jinxin Xie, Panpan Tong

**Affiliations:** ^1^ College of Veterinary Medicine, Xinjiang Agricultural University, Urumqi, China; ^2^ Xinjiang Key Laboratory of New Drug Research and Development for Herbivores, Urumqi, China; ^3^ Key Lab Animal Bacteriology, Ministry of Agriculture, College of Veterinary Medicine, Nanjing Agricultural University, Nanjing, China; ^4^ Ili Kazakh Autonomous Prefecture General Animal Husbandry Station, Xinjiang Uighur Autonomous Region, Yining, China

**Keywords:** avian pathogenic *Escherichia coli*, multidrug-resistant, bacteriophage, characteristics, broad-spectrum

## Abstract

**Introduction:**

The widespread misuse of antibiotics has accelerated the emergence of multidrug-resistant bacterial strains, presenting a major threat to global public health. Bacteriophages (phages), owing to their host-specific lytic activity and self-replicating nature, have emerged as promising alternatives or adjuncts to conventional antibiotic therapies.

**Methods:**

In this study, a lytic phage targeting avian pathogenic Escherichia coli (APEC) was isolated from farm wastewater. The phage’s morphological characteristics, host range, optimal multiplicity of infection (MOI), one-step growth curve, pH stability, thermal stability, chloroform sensitivity, and in vitro antibacterial activity were determined. Subsequently, the therapeutic efficacy of the phage was evaluated in a pigeon model.

**Results:**

In this study, we isolated and characterized a lytic phage, designated vB_EcoM_P3322, from farm wastewater targeting APEC. Transmission electron microscopy classified vB_EcoM_P3322 within the Myoviridae family. The phage exhibited broad lytic activity against five Escherichia coliserotypes (O8:H10, O15:H18, O51:H20, O149:H20, and O166:H6). Optimal biological parameters included a multiplicity of infection (MOI) of 1, a latent period of 10 minutes, an 80-minute burst period, and a burst size of 252 PFUs/cell. vB_EcoM_P3322 maintained stable lytic activity across a pH range of 5–9 and temperatures from 4°C to 50°C, although it was sensitive to chloroform. In vitro, the phage effectively suppressed bacterial growth within 6 hours at MOIs of 0.1, 1, and 10. Whole-genome sequencing revealed a 151,674 bp double-stranded DNA genome encoding 279 predicted open reading frames. No virulence factors, toxin genes, antibiotic resistance genes, or lysogeny-related elements were identified, affirming its safety for therapeutic application. Phylogenetic analysis indicated 98.44% nucleotide identity (97% coverage) with phage vB_EcoM_Ro121c4YLVW (GenBank: NC_052654), suggesting a close evolutionary relationship. In a pigeon infection model, vB_EcoM_P3322 treatment significantly improved survival and reduced histopathological damage in the liver and spleen. Metagenomic analysis of duodenal contents revealed a marked reduction (P < 0.01) in E. coli abundance in the treatment group, indicating selective pathogen clearance and modulation of gut microbiota.

**Discussion:**

In summary, vB_EcoM_P3322 displays broad-spectrum lytic activity, robust environmental stability, potent antibacterial efficacy both in vitro and in vivo, and a safe genomic profile. These attributes support its potential as a novel biocontrol agent for managing APEC infections in poultry farming.

## Introduction

*Escherichia coli*, a ubiquitous bacterium found in the intestinal tracts of warm-blooded animals, has a dual nature: while commensal strains aid digestion, pathogenic variants pose severe threats to both human and animal health (Riley, 2020). Among these, avian pathogenic *E.coli* (APEC) is responsible for systemic poultry disease, resulting in devastating conditions, such septicemia, pericarditis, and perihepatitis, leading to substantial economic losses in the global poultry industry (Joseph et al., 2023; Karami et al., 2024).

Decades of reliance on antibiotics, accompanied by overuse and inappropriate use in agriculture, has led to the development of multidrug-resistant (MDR) APEC strains, eroding the therapeutic efficacy of the drugs (Poirel et al., 2018; Kathayat et al., 2021). Changes in global regulations, including the European Union ban on antibiotic growth promoters, underscore the urgency of developing sustainable alternatives that circumvent resistance while ensuring food safety and animal welfare (Tang et al., 2023; Medina and Pieper, 2016).

A potential solution is the use of bacteriophages (phages). Phages can target pathogens precisely and proliferate at infection sites, with negligible impact on the host microbiota or eukaryotic cells (Altamirano and Barr, 2021; Mboowa, 2023; Norambuena et al., 2025). However, the narrow host specificity of phages necessitates the development of novel solutions, such as the identification of broad-spectrum phages and optimization of phage cocktails (Yang et al., 2020; Abedon et al., 2021).

To bridge this gap, the present study isolated and characterized vB_EcoM_P3322, a novel, environmentally sourced lytic phage with potent activity against APEC. The results of assessments of its biological traits, genomic safety, and *in vivo* efficacy suggest its potential as a targeted, antibiotic-free intervention. The study findings not only advance phage biology but also suggest a method for sustainable poultry production.

## Materials and methods

### Bacterial strains and growth conditions

A total of 97 *Escherichia coli* strains were isolated from the diarrhea of pigeons, chickens, and geese in Xinjiang. The isolates were obtained using selective media followed by PCR confirmation. These bacteria were used for phage isolation and determination of lytic profiles. All bacterial strains were cultured in Luria-Bertani (LB, Qingdao Hope Bio-Technology Co., Ltd.) broth at 37°C.

### Isolation and purification of phage

Fresh sewage samples were collected from the drainage outlet of a pigeon farm in Hotan, China, and transported to the laboratory under refrigerated conditions (4°C), where they were stored at the same temperature for short-term use. For phage isolation, 20 mL of the sewage sample was combined with 10 mL of LB broth, followed by the addition of 3 mL of the host strain 3-32-2 a MDR APEC clinical isolate obtained from a pigeon with diarrhea. The host strain (OD_600_ = 0.5–0.6) belonged to serotype O166 and sequence type ST646, and harbored both virulence genes (*irp2*, *fyuA*, *fimH*, *iroN*) and resistance genes (*bla*_TEM-20_, *tet*(A), *sul1*). The mixture was incubated at 37°C with shaking at 180 rpm for 12–16 hours to enrich for phages.

After incubation, the culture was centrifuged at 11,000 rpm for 10 minutes, and the resulting supernatant was filtered through a 0.22 µm sterile membrane to obtain the phage-containing lysate. This lysate was then mixed with an equal volume of *Escherichia coli* 3-32-2 and molten semi-solid agar (0.5% agarose), and the mixture was overlaid onto LB agar plates using the double-layer agar technique. Plates were incubated at 37°C for 12–18 hours. Individual clear plaques were isolated and subjected to multiple rounds of purification using the same double-layer agar method until homogeneous and well-defined plaques were achieved. The purified phage was subsequently stored at 4°C for further characterization.

### Phage morphology

The phage morphology was examined using transmission electron microscopy (TEM). In brief, purified high-titer phage particles were deposited onto a copper grid and negatively stained with 2% phosphor tungstic acid (PTA). After gently removing excess liquid, the grid was observed using a HT7800 TEM (Hitachi, Ltd., Japan).

### Determination of phage lytic spectrum

The host range of the phage was determined by spot assay with minor modifications from the previously reported method by Asghar et al., 2022. Briefly, 100 µL of each *Escherichia coli* suspension was mixed with 8 mL of LB semi-solid medium (containing 0.5% agar) and promptly overlaid onto LB agar plates. Once the overlay solidified, 5 µL of the phage suspension was spotted onto the surface of each bacterial lawn. The plates were incubated overnight at 37°C, and the formation of clear plaques was used as an indicator of phage-induced lysis.

### Multiplicity of infection assay

The infection kinetics were analyzed following the method previously reported by Li et al., 2019 with slight modifications. The host strain 3-32-2 was mixed with serial dilutions of phage suspensions (ranging from 1×10^6^ to 1×10¹^0^ PFU/mL) at MOI values of 0.01, 0.1, 1, 10, and 100. The mixtures were incubated at 37°C with shaking at 180 rpm for 6 hours. Following incubation, samples were filtered through 0.22 µm membranes to remove bacterial cells. Phage titers were then determined using the standard double-layer agar assay, and plates containing 30 to 300 phage spots were selected for counting. The phage potency (PFU/mL) was calculated as the number of phage spots × 10 × inverse of dilution. The experiments were performed independently three times.

### Phage one-step growth curve assay

The phage replication cycle were examined with slight modifications based on the method described previously by Zhou et al., 2022. In brief, phages were mixed with the host strain at a MOI of 1 and incubated at 37°C for 10 minutes to allow adsorption. The mixture was then centrifuged at 11,000 rpm for 10 minutes to separate the unadsorbed phages, which were removed by discarding the supernatant. The resulting pellet was washed twice with Luria-Bertani (LB) broth and resuspended in 10 mL of pre-warmed LB medium. The culture was incubated at 37°C with shaking at 180 rpm, and samples were collected at 10-minute intervals over a 2-hour period. Phage titers at each time point were quantified using the standard double-layer agar method. All experiments were conducted in triplicate. The experiments were performed independently three times.

### Thermal and pH stability

Phage thermal stability was assessed by incubating phage filtrates at various temperatures (4, 25, 37, 40, 50, 60, 70, and 80°C) for 1 hour. Samples were collected at 20, 40, and 60 minutes during incubation, and phage titers were determined using the standard double-layer agar method. All temperature stability tests were conducted in triplicate. The experiments were performed independently three times.

For pH stability evaluation, phage filtrates were mixed with LB broth adjusted to pH values ranging from 2 to 13 using HCl for acidic conditions and NaOH for alkaline conditions. The mixtures were incubated at 37°C for 1 hour, after which phage titers were measured using the double-layer agar assay. Each pH condition was tested in three independent replicates. The experiments were performed independently three times.

### Chloroform sensitivity test

The phage filtrate was treated with chloroform at a final concentration of 10% (v/v), thoroughly mixed, and incubated in a water bath at 37°C for 30 minutes. After incubation, phage titers were assessed using the standard double-layer agar plaque assay. All experiments were conducted in triplicate to ensure statistical reliability. The experiments were performed independently three times.

### Inhibition of planktonic bacterial cells by phages *in vitro*

The phage-mediated growth inhibition was evaluated with modifications to the previously described method by Yao et al., 2023. Host bacteria were co-cultured with phages at MOIs of 0.1, 1, and 10 in LB broth at 37°C with shaking at 180 rpm for 12 hours. A bacterial culture without phage treatment served as the negative control. Bacterial growth dynamics were monitored by measuring optical density at 600 nm (OD_600_) (BioTek Synergy HTX, USA) at 2-hour intervals. All experiments were conducted in triplicate. The experiments were performed independently three times.

### Phage genome sequencing and analysis

Phage genomic DNA was extracted using the Rapid Bacteriophage DNA Extraction Kit (Geneaid, Taiwan), and its concentration was measured with a NanoDrop spectrophotometer (Bio-Rad, USA). Whole-genome sequencing was carried out using the Illumina NovaSeq™ platform. Raw sequencing reads were assembled into a complete genome using SPAdes v3.5.0, followed by gap closure with GapFiller v1.11 and genome refinement using PrInSeS-G v1.0.0. The assembled genome was analyzed for the presence of virulence factors using the Virulence Factor Database (VFDB, http://www.mgc.ac.cn/VFs/main.htm). Antibiotic resistance genes using the Comprehensive Antibiotic Resistance Database (CARD, https://card.mcmaster.ca/). tRNA genes were predicted using tRNAscan-SE (https://lowelab.ucsc.edu/tRNAscan-SE/). Genome visualization was performed using the CGView Server (http://cgview.ca/). Comparative genomics and phylogenetic analysis were conducted using NCBI BLASTN (http://blast.ncbi.nlm.nih.gov). A neighbor-joining phylogenetic tree was constructed to compare the complete genomes of the phages with those of their 10 closest 10 *Phapecoctavirus* phage relatives, using MEGA v11 with 1000 bootstrap replications.

### Efficacy of phage therapy for APEC infection in pigeon model

The therapeutic efficacy of phage treatment against APEC infection in pigeons was evaluated using an oral gavage administration protocol. Phage suspensions were administered at three different timepoints relative to APEC infection 3 hours before (prophylactic), at the time of infection (concurrent), and 3 hours after (therapeutic) to assess both preventive and curative effects. A total of forty-eight 30-day-old commercial white-feathered meat pigeons were randomly assigned to six groups (n = 8 per group): three treatment groups (prophylactic, concurrent, and therapeutic), an APEC-only group, a phage-only group, and a control group. APEC strain 3-32-2 was delivered via intraperitoneal injection (4 mL of 1×10^9^ CFU/mL), while phage vB_EcoM_P3322 was administered by oral gavage (4 mL of 1×10^9^ PFU/mL). Pigeons were monitored daily for 7 days to determine survival rates. Body weight and food intake were recorded throughout the experiment. At the end of the study, blood samples were collected from the wing veins, and serum was separated and stored at –20°C for subsequent analysis. Surviving pigeons were humanely euthanized on day 7 post-infection. Under sterile conditions, organs including the heart, liver, spleen, lungs, kidneys, brain, and duodenum were harvested and fixed in 4% paraformaldehyde for histopathological examination. Tissue sections were embedded, sectioned, and stained with hematoxylin and eosin (H&E) for microscopic analysis. To evaluate the impact of treatment on gut microbiota, duodenal samples from three randomly selected pigeons per group were subjected to metagenomic sequencing. Sequencing was carried out by Beijing Novogene Co., Ltd. Raw sequencing data were preprocessed using fastp (https://github.com/OpenGene/fastp) to remove low-quality reads and generate high-quality clean data. In cases of potential host contamination, reads were aligned to the host genome using Bowtie2 (http://bowtie-bio.sourceforge.net/bowtie2/index.shtml), and any reads matching the host were filtered out. Using For taxonomic annotation, the remaining high-quality reads were aligned to the Micro_NR database using DIAMOND (https://github.com/bbuchfink/diamond/) to identify metagenomic species. Principal coordinate analysis (PCoA) was performed using the ade4 package to assess differences in microbial community structure among groups. To identify species with significant differences between groups, both MetaGenomeSeq and Linear Discriminant Analysis Effect Size (LEfSe) analyses were employed. MetaGenomeSeq was used to perform statistical testing at various taxonomic levels, generating p-values for intergroup comparisons. LEfSe analysis was conducted using LEfSe software, with a linear discriminant analysis (LDA) score threshold of >2.0 to identify taxa with significant differential abundance.

### Statistical analyses

Statistical analyses were performed using GraphPad Prism and R software. Comparisons between groups were conducted using the T-test. Statistical significance was denoted as follows: * *P* < 0.05 (significant), ** *P* < 0.01 (highly significant), and *** *P* < 0.001 (extremely significant).

## Results

### Isolation, purification, and morphology of phage

**Figure 1:**
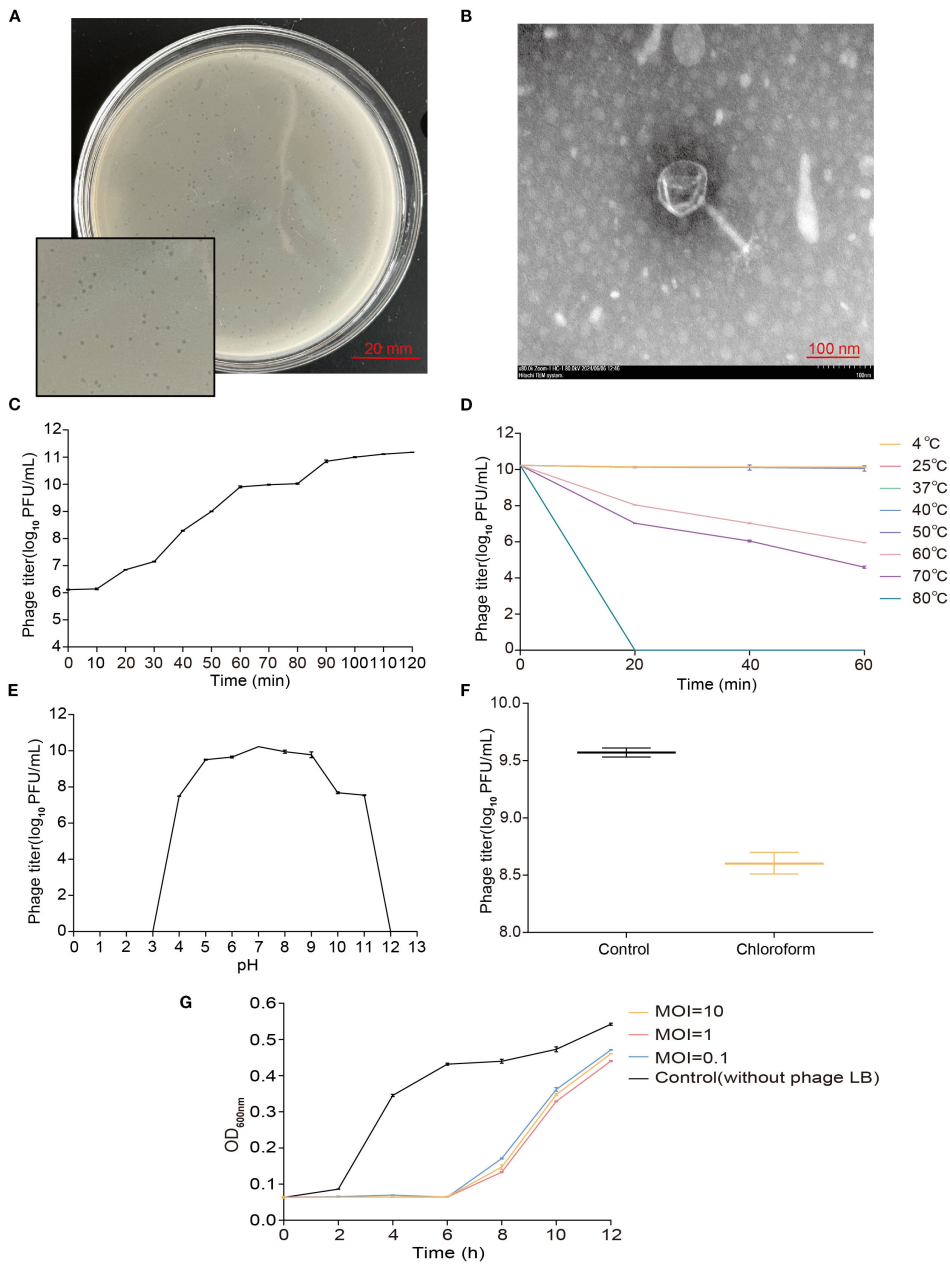
Biological characteristics of phage vB_EcoM_P3322. **(A)** Plaque morphology. **(B)** TEM morphology (Scale bar = 100 nm). **(C)** One-step growth curve (latent period and burst size). **(D)** Thermal stability. **(E)** pH tolerance. **(F)** Chloroform sensitivity. **(G)** In vitro inhibition curve.

Phage vB_EcoM_P3322 was isolated and purified from sewage using APEC strain 3-32-2 as the host. On double-layer agar plates, it produced clear, circular plaques approximately 1 mm in diameter with well-defined edges (**Figure 1A**).

TEM analysis revealed that vB_EcoM_P3322 has an icosahedral head measuring approximately 91 nm in diameter and a contractile tail sheath around 105 nm in length (**Figure 1B**).

The phage demonstrated lytic activity against 65 of the 97 *Escherichia coli* isolates derived from various animals, including pigeon (60/91), goose (3/3), and chicken (2/3). Whole-genome sequencing of five representative susceptible strains (based on different ERIC-PCR fingerprinting patterns and strong lytic ability) led to the identification of five predicted serotypes: O8:H10, O15:H18, O51:H20, O149:H20, and O166:H6 (**Table 1**).

**Table 1:** The lytic spectrum of phage vB_EcoM_P3322.

Animal	Bacteria	Serotype	Major virulence genes	Major drug resistance genes
Pigeon	*Escherichia coli*	O8:H10	*irp2*, *fyuA*, *fimH*, *vat*, *iroN*	*bla*_TEM-235_, *sul1*, *tet*(A)
Pigeon	*Escherichia coli*	O15:H18	*irp2*, *fyuA*, *fimH*, *iroN*	*bla*_TEM-20_, *tet*(A), *sul1*
Pigeon	*Escherichia coli*	O149:H20	*fimH*, *iroN*	*bla*_EC-8_
Pigeon	*Escherichia coli*	O166:H6	*irp2*, *fyuA*, *fimH*, *iroN*	*bla*_TEM-235_, *aph*(6)*-Id*, *tet*(A), *floR*
Goose	*Escherichia coli*	O51:H20	*irp2*, *fyuA*, *fimH*, *iutA*, *iroN*, *iucD*	*bla*_TEM-20_, *qnrS1*, *tet*(A)

The host strain 3-32-2 was infected with phage vB_EcoM_P3322 at varying MOIs to determine the phage titer and identify the optimal MOI. The highest phage titer was observed at an MOI of 1, indicating this as the optimal infection ratio (**Table 2**).

**Table 2:** Optimal MOI of phage vB_EcoM_P3322.

MOI	Phage tite (PFU/mL)	Bacterial concentration (CFU/mL)	Phage titers after incubation (CFU/mL)
100	1.4×10^10^	1.4×10^8^	7.0×10^10^
10	1.4×10^9^	1.4×10^8^	1.4×10^11^
1	1.4×10^8^	1.4×10^8^	3.3×10^11^
0.1	1.4×10^7^	1.4×10^8^	6.5×10^9^
0.01	1.4×10^6^	1.4×10^8^	4.0×10^8^

One-step growth curve analysis (**Figure 1C**) revealed that phage vB_EcoM_P3322 has a latent period of approximately 10 minutes, followed by a burst period of 80 minutes, with an average burst size of 252 PFUs/cell (Burst size = phage titer at the end of lysis/number of host strains at the start of infection). The phage enters the stationary phase at around 90 minutes. Stability assessments under different environmental conditions showed that vB_EcoM_P3322 remains thermally stable across a broad temperature range (4–50°C), as shown in **Figure 1D**. However, its titer declined progressively at temperatures above 50°C and was completely inactivated after 20 minutes at 80°C. In terms of pH stability, the phage remained active between pH 5 and 9 (**Figure 1E**). A sharp decline in activity was observed at pH 4, 10, and 11, and complete inactivation occurred at extreme pH values of 2, 12, and 13. Treatment with 10% chloroform at 37°C for 30 minutes resulted in a ~1 log_10_ PFU/mL reduction in phage titer, indicating partial sensitivity to chloroform (**Figure 1F**). To evaluate its antibacterial efficacy *in vitro*, strain 3-32-2 was exposed to phage vB_EcoM_P3322 at MOIs of 0.1, 1, and 10. In addition, compared to the untreated control, the phage effectively suppressed bacterial growth for up to 6 hours at all tested MOIs (**Figure 1G**). These characteristics underscore its potential as a stable and potent biocontrol agent for managing APEC infections in poultry.

### Genome, comparative genomics and phylogenetic analysis of phage vB_EcoM_P332

**Figure 2:**
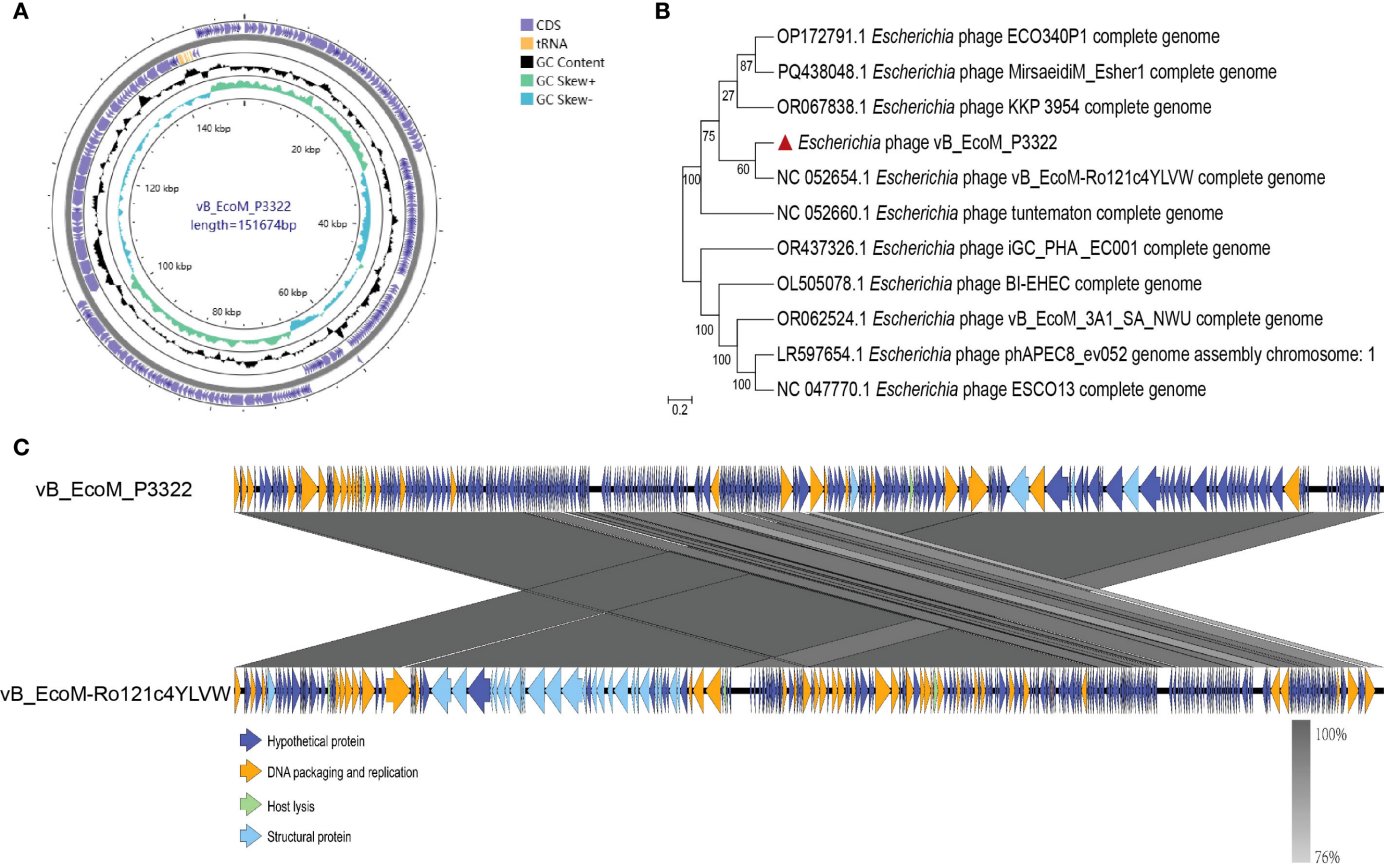
Genomic characterization of phage vB_EcoM_P3322. **(A)** The whole genome map. **(B)** The whole-genome evolutionary tree. **(C)** Linear comparative analysis between the complete genes of phage vB_EcoM_P3322 and vB_EcoM-Ro121c4YLVW. The color shadow in the middle shaded area indicates the degree of homology. The arrows indicate the open reading boxes transcribed to the right or left.

Whole-genome sequencing of phage vB_EcoM_P3322 revealed a genome size of 151,674 base pairs with a GC content of 39.14% (**Figure 2A**). Genomic annotation identified 279 open reading frames (ORFs), of which only 36 (12.9%) were assigned known functions, while the remaining 243 (87.1%) were annotated as hypothetical proteins. The functionally annotated ORFs were categorized into three groups: DNA packaging and replication proteins, structural proteins, and host lysis proteins. Additionally, ten tRNA genes were predicted within the genome. Importantly, no lysogeny-related genes were detected, indicating that vB_EcoM_P3322 is a strictly lytic (virulent) phage. In addition, screening against the Virulence Factor Database (VFDB) and the Comprehensive Antibiotic Resistance Database (CARD) confirmed the absence of virulence and antibiotic resistance genes, supporting the genomic safety of vB_EcoM_P3322 for potential clinical or veterinary applications.

BLAST analysis revealed that the genome of phage vB_EcoM_P3322 shares significant similarity with multiple phages isolated globally, suggesting complex evolutionary relationships among them. Phylogenetic analysis grouped these phages into two major clades (**Figure 2B**), with vB_EcoM_P3322 clustering closely with *Escherichia coli* phage vB_EcoM_Ro121c4YLVW, showing 98.44% sequence identity and 97% coverage, indicating a close genetic relationship. Comparative genomic visualization using Easyfig 2.2.5 (**Figure 2C**) showed that vB_EcoM_P3322 and vB_EcoM_Ro121c4YLVW share high homology (>76%) across most functional genomic regions, although with opposite regional alignments in some areas. Based on its genomic characteristics, vB_EcoM_P3322 is classified under the class *Caudoviricetes* and belongs to the genus *Phapecoctavirus*.

### Therapeutic efficacy of phage vB_EcoM_P3322

**Figure 3:**
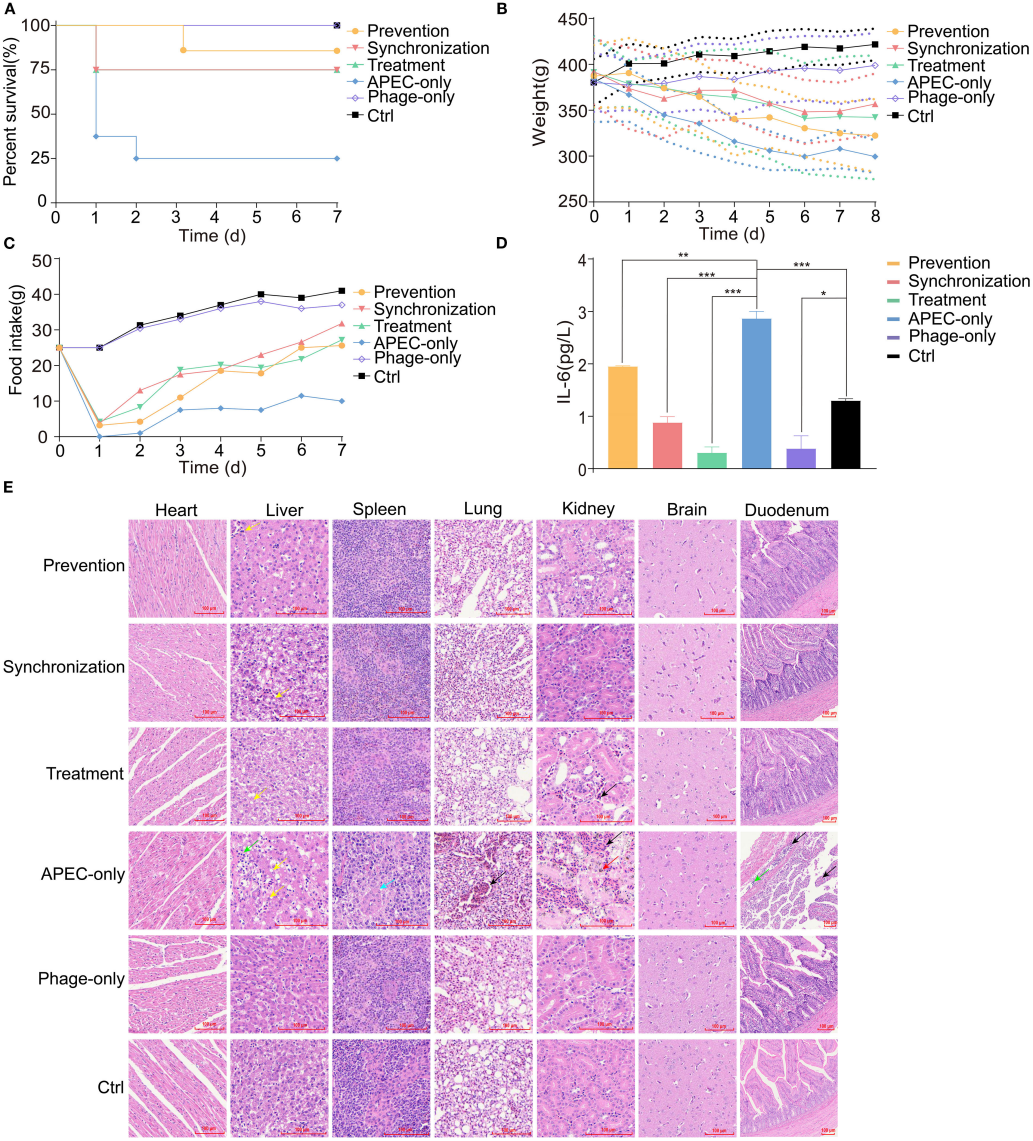
Therapeutic efficacy of phage vB_EcoM_P3322 in APEC-infected squab. **(A)** The survival rate of young pigeons. **(B)** The weight of the young pigeons. **(C)** The food intake of young pigeons. **(D)** The content of IL-6 in serum * *P* < 0.05 (significant), ** *P* < 0.01 (highly significant), and *** *P* < 0.001 (extremely significant). **(E)** Histopathological examination of the heart, liver, spleen, lungs, kidneys, brain and duodenum of the young pigeons 7 days after infection. Black arrow: Indicates hemorrhage. Yellow arrow: Indicates hepatocellular swelling with abundant lipid droplets in the cytoplasm. Green arrow: Indicates mild inflammatory cell infiltration. Blue arrow: Indicates vascular endothelial swelling and reduced size of lymphoid follicles. Red arrow: Indicates deposition of hemosiderin granules. * *P* < 0.05 (significant), ** *P* < 0.01 (highly significant), and *** *P* < 0.001 (extremely significant).

The protective efficacy of phage vB_EcoM_P3322 against APEC infection was evaluated in a young pigeon model. The phage was administered via oral gavage at three different timepoints relative to APEC infection: phage administered 3 hours before APEC challenge (prevention group), phage administered simultaneously with APEC challenge (synchronization group), and phage administered 3 hours after APEC challenge (treatment group). The survival rate in the APEC-only group was 25%, while pigeons treated with phage 3 hours before infection showed the highest survival rate at 87.5%. Survival rates in the 0 h and 3 h post-infection treatment groups were both 75% (**Figure 3A**), indicating that vB_EcoM_P3322 significantly improved survival following APEC challenge. Additionally, phage-treated groups exhibited higher body weights compared to the APEC-only group, suggesting that phage therapy helped mitigate weight loss associated with APEC infection (**Figure 3B**). Feed intake decreased in all infected groups one day post-infection but gradually recovered in the phage-treated groups (the prevention, synchronization, and treatment groups), aligning more closely with the uninfected control group over time (**Figure 3C**). Serum levels of interleukin-6 (IL-6), measured via enzyme-linked immunosorbent assay (ELISA), were significantly elevated in the APEC-only group compared to the control (**Figure 3D**). In contrast, IL-6 levels were significantly reduced (*P* < 0.05) in all three phage-treated groups, indicating that vB_EcoM_P3322 treatment effectively alleviated the inflammatory response induced by APEC infection.

The host strain 3-32-2 did not induce any observable pathological changes in the heart or brain tissues. In contrast, significant tissue damage was observed in the APEC-infected group. Liver histology revealed hepatocellular swelling with numerous lipid droplets of varying sizes, indicative of steatosis. In the spleen, endothelial swelling of the central artery was noted, along with a reduction in the size of surrounding lymphoid follicles. Lung tissues showed extensive hemorrhage, while kidney sections exhibited widespread bleeding and hemosiderin granule deposition in the tubulointerstitium. Duodenal tissue displayed marked submucosal edema, enlarged interstitial spaces, bleeding, and infiltration of inflammatory cells, with hemorrhage observed in the intestinal villi. In comparison, phage treatment at all three timepoints (the prevention, synchronization, and treatment groups) resulted in a noticeable reduction of lipid droplets in the liver and partial alleviation of pathological changes in the spleen, lungs, kidneys, and duodenum. These tissue structures more closely resembled those of healthy, uninfected pigeons. Collectively, these findings suggest that phage vB_EcoM_P3322 can effectively mitigate multi-organ damage caused by APEC infection (**Figure 3E**).

**Figure 4:**
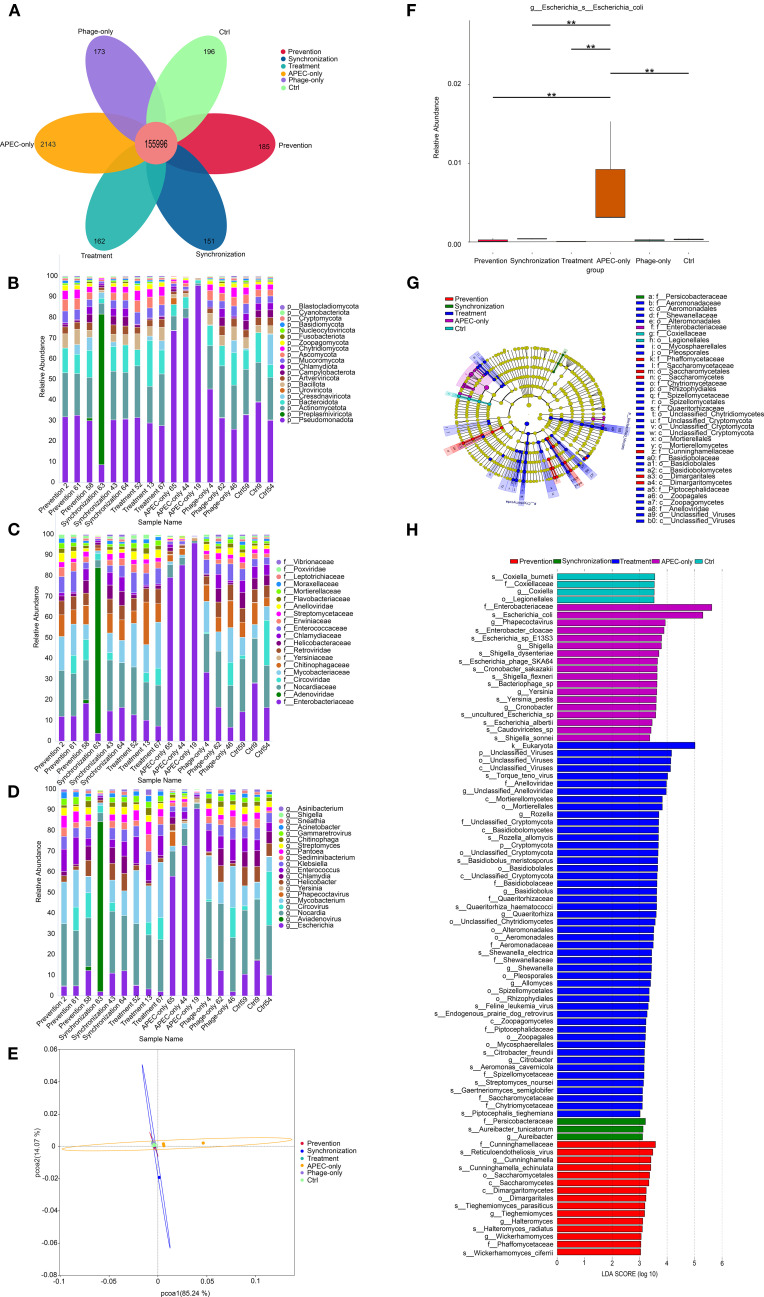
Duodenal microbiota modulation by phage treatment. **(A)** Petal diagram. Bar chart analysis of the composition of the top 20 microbial communities at the phylum, family and genus levels **(B–D)**. **(E)** Principal coordinate analysis (PCOA) based on the bray-Curtis distance calculated from the OTU abundance matrix. **(F)** The MetaGenome Seq method conducts hypothesis testing on the functional abundance data between groups using the fitFeatureModel function to obtain the p value. Finally, the functions with significant differences were screened based on the p value, and the abundance distribution box plot of the differential functions between groups was drawn (the bars at the level of asterisons (*) indicated the degree of significant differences (*P value<0.05, **P value<0.01). **(G)** LEfSe discriminant analysis of multi-level species differences from the phylum to the species level. **(H)** Linear discriminant analysis (LDA) histogram from gate to seed level; The LDA value distribution bar chart shows the species whose LDA Score is greater than the set value of 2, that is, the biomarkers with statistical differences between groups. The length of the bar chart represents the degree of influence of the different species (that is, the LDA Score).

Petal plot analysis (**Figure 4A**) revealed a total of 159,006 operational taxonomic units (OTUs) identified in the duodenal microbiota of the young pigeons, with 155,996 OTUs shared across all groups. Unique OTUs were distributed as follows: 185 in the prevention group, 151 in the synchronization group, 162 in the treatment group, 2,143 in the APEC-only group, 173 in the phage-only group, and 196 in the control group. These results indicate notable differences in gut microbial composition among the six experimental groups. Notably, APEC infection led to an increase in distinct OTUs, whereas phage treatment effectively reduced the abundance of these characteristic OTUs, suggesting a modulatory effect on the intestinal microbiota.

To gain deeper insights into the alterations in intestinal microbiota composition, we analyzed the dominant taxa at the phylum, family, and genus levels (**Figures 4B-D**). At the phylum level, *Pseudomonadota*, *Actinobacteriota*, and *Bacteroidota* were predominant. Notably, *Pseudomonadota* showed a marked increase in relative abundance in the APEC-infected group, becoming the dominant phylum (**Figure 4B**). At the family level (**Figure 4C**), *Enterobacteriaceae* abundance was significantly elevated in the APEC group compared to the other groups. At the genus level (**Figure 4D**), *Escherichia coli* was substantially more abundant in the APEC-infected pigeons. However, its abundance significantly declined in all three phage-treated groups, indicating the effectiveness of the phage in reducing *Escherichia coli* proliferation within the gut microbiota.

PCoA based on Bray-Curtis distance at the OTU level (**Figure 4E**) revealed that the first two principal components accounted for 85.24% (PC1) and 14.07% (PC2) of the variance, respectively. The APEC-infected group was clearly separated from the other groups, while the prevention, synchronization, treatment, phage-only, and control groups clustered closely together. This indicates that phage treatment helped maintain microbial community structure and mitigated the disruption caused by *Escherichia coli* infection.

Further analysis using the MetaGenomeSeq method (**Figure 4F**) showed that the abundance of *Escherichia coli* was significantly reduced (*P* < 0.01) in the phage-treated groups (prevention, synchronization, and treatment) as well as in the control group compared to the APEC group, confirming the phage’s efficacy in suppressing *Escherichia coli* overgrowth.

Thirty-seven OTUs were analyzed using LEfSe, and the results indicated that there were significant differences among these OTUs from the gate to the species level (**Figure 4G**). Further analysis (**Figure 4H**) identified 89 taxa with statistically significant differences among the prevention, synchronization, treatment, APEC-only, and control groups. These included 1 kingdom, 2 phyla, 7 classes, 15 orders, 15 families, 17 genera, and 32 species. The findings indicate that *Escherichia coli* infection in young pigeons significantly alters the composition of the duodenal microbiota. In the APEC-infected group, the relative abundance of *Enterobacteriaceae* and *Escherichia coli* in the duodenum was notably elevated, underscoring the microbial imbalance caused by infection.

Duodenal metagenomic sequencing analysis (**Figures 4B-D**) revealed the presence of Pigeon Adenovirus and Pigeon Circovirus (PiCV) in some meat pigeons. Despite this, the affected pigeons exhibited normal behavior, feed and water intake, and showed no typical clinical signs such as lethargy, diarrhea, or respiratory distress. Histopathological examination also revealed no significant damage to intestinal or immune tissues, confirming that these pigeons were asymptomatic carriers of both adenovirus and circovirus.

## Discussion

The phage vB_EcoM_P3322 isolated in this study exhibited a broad host range, effectively lysing 65 out of 97 animal-derived *Escherichia coli* strains maintained in our laboratory (67%), encompassing at least five O serotypes, including several clinically multidrug-resistant isolates. This lytic spectrum is markedly broader than that of several previously reported phages. For example, phage MJ1, which targets multidrug-resistant *Escherichia coli*, lysed only 3 out of 32 strains (9.4%) (Jamal et al., 2015), while phage fEg-Eco19 demonstrated an even lower lysis rate of just 2 out of 137 strains (1.5%) (Badawy et al., 2022). These comparisons highlight the superior lytic potential of vB_EcoM_P3322, especially for combating drug-resistant strains. The enhanced host range of vB_EcoM_P3322 may be attributed to its efficient recognition of diverse bacterial surface receptors or its evolutionary acquisition of broader host adaptability. Variations in lytic profiles among phages are likely influenced by factors such as environmental origin, host strain diversity, and the extent of phage-host co-evolution. Future investigations should focus on characterizing the receptor-binding proteins of vB_EcoM_P3322 to elucidate the molecular basis of its broad host specificity and to assess its practical application in controlling animal-derived, drug-resistant *Escherichia coli* infections.

The phage vB_EcoM_P3322 exhibited demonstrated outstanding biological properties, including a short latent period of 10 minutes and a high burst size of 252 PFUs/cell, indicating its strong infectivity and replication efficiency. This rapid lytic activity enables it to eliminate host bacteria more effectively within a short time frame, enhancing its potential as a powerful biocontrol agent (Alexyuk et al., 2022; Wang et al., 2022). Regarding environmental adaptability, vB_EcoM_P3322 exhibited excellent thermal and pH stability. These findings are consistent with previously reported optimal conditions for phage activity (30–50°C, pH 5–9) (Liu et al., 2024; Zhang et al., 2025), suggesting that vB_EcoM_P3322 is well-suited to withstand the dynamic conditions of the animal gastrointestinal tract. Importantly, the stability of phages under environmental stress is a critical factor influencing their effectiveness in real-world applications. The demonstrated tolerance of vB_EcoM_P3322 to variable temperatures and pH, particularly its resilience in the gastrointestinal environment strongly supports its potential use in controlling drug-resistant *Escherichia coli* infections in livestock.

The genomic characterization of phage vB_EcoM_P3322 offers strong evidence supporting its biological safety for therapeutic use. Comprehensive functional annotation showed that the genome lacks any known virulence factors, toxin genes, antibiotic resistance determinants, or lysogeny-associated elements. This absence significantly minimizes the risk of horizontal gene transfer during application, providing a molecular assurance of its biosafety (Balcazar, 2014; Salmond and Fineran, 2015). A total of 10 tRNA genes were identified. The presence of tRNA genes holds taxonomic relevance, as studies have shown that these genes are typically found in virulent phages but are largely absent in temperate phages (Bailly-Bechet et al., 2007; Lomeli‐Ortega and Balcázar, 2024). The detection of tRNA genes in vB_EcoM_P3322 further supports its classification as a strictly lytic phage, indicating it does not mediate lysogenic conversion, an important consideration for safe therapeutic use. Together, these genomic features confirm that vB_EcoM_P3322 possesses both high biocontrol potential and meets essential genetic safety criteria, reinforcing its suitability as a candidate for controlling drug-resistant *Escherichia coli* infections in veterinary settings.

The therapeutic evaluation of phage vB_EcoM_P3322 in a young pigeon model of *Escherichia coli* infection yielded promising results. Notably, the prophylactic group exhibited the highest survival rate, suggesting that pre-establishing a sufficient phage titer in the host may provide enhanced protection by enabling immediate response to subsequent bacterial invasion. Histopathological examinations further confirmed the therapeutic benefits of phage vB_EcoM_P3322. Tissues from the liver, spleen, lungs, kidneys, and duodenum of phage-treated pigeons exhibited milder pathological changes compared to those from the untreated infected group, correlating with improved survival outcomes. Additionally, duodenal metagenomic sequencing revealed a significant reduction in the relative abundance of *Escherichia coli* in all phage-treated groups (prevention, synchronization, and treatment). This finding is particularly important, as maintaining a balanced intestinal microbiota is crucial for the host’s physiological functions and overall health (Zhang et al., 2015; Wang et al., 2020).

This study identifies phage vB_EcoM_P3322 as a promising biotherapeutic candidate for combating *Escherichia coli* infections, particularly those caused by APEC. The phage exhibited strong antibacterial activity, with notable effectiveness when administered prior to bacterial exposure. The highest survival rates were observed in the prophylactic treatment group, suggesting that pre-emptive phage administration can establish a protective phage population within the host, thereby enhancing resistance to subsequent bacterial infection. These findings underscore the potential of vB_EcoM_P3322 not only as a therapeutic agent but also as a preventive intervention in both clinical veterinary applications and agricultural production systems. Its use could be especially valuable in high-risk settings such as poultry farms, where early phage administration might reduce infection incidence, limit antibiotic use, and mitigate the spread of multidrug-resistant *Escherichia coli* strains. Further investigation into optimal dosing strategies and delivery methods will support its development as a practical and scalable tool for pathogen control in livestock.

## Data Availability

The datasets presented in this study can be found in online repositories. The names of the repository/repositories and accession number(s) can be found below: https://www.ncbi.nlm.nih.gov/genbank/, PV191315 https://www.ncbi.nlm.nih.gov/sra/, PRJNA1254409.
